# Food Poisonings by Ingestion of Cyprinid Fish

**DOI:** 10.3390/toxins6020539

**Published:** 2014-01-28

**Authors:** Manabu Asakawa, Tamao Noguchi

**Affiliations:** 1 Graduate School of Biosphere Science, Hiroshima University, Higashi-Hiroshima, Hiroshima 739-8528, Japan; 2 Division of Medical Nutrition, Tokyo Healthcare University Graduate School, 3-11-3, Setagaya, Setagaya-ku, Tokyo 154-8568, Japan; E-Mail: t-noguchi@thcu.ac.jp

**Keywords:** cyprinid fish, gallbladder, bile alcohol, Thin layer chromatography, acute renal failure, acute dysfunction of liver, FAB mass spectrometry, IR spectrometry, NMR spectrometry

## Abstract

Raw or dried gallbladders of cyprinid fish have long been ingested as a traditional medicine in the Asian countries, particularly in China, for ameliorating visual acuity, rheumatism, and general health; however, sporadic poisoning incidences have occurred after their ingestion. The poisoning causes complex symptoms in patients, including acute renal failure, liver dysfunction, paralysis, and convulsions of limbs. The causative substance for the poisoning was isolated, and its basic properties were examined. The purified toxin revealed a minimum lethal dose of 2.6 mg/20 g in mouse, when injected intraperitoneally*.* The main symptoms were paralysis and convulsions of the hind legs, along with other neurological signs. Liver biopsy of the euthanized mice clearly exhibited hepatocytes necrosis and infiltration of neutrophils and lymphocytes, suggesting the acute dysfunction of the liver. Blood tests disclosed the characteristics of acute renal failure and liver injury. Infrared (IR) spectrometry, fast atom bombardment (FAB) mass spectrometry, and ^1^H- and ^13^C-nuclear magnetic resonance (NMR) analysis indicated, a molecular formula of C_27_H_48_O_8_S, containing a sulfate ester group for the toxin. Thus, we concluded that the structure of carp toxin to be 5α-cyprinol sulfate (5α-cholestane-3α, 7α, 12α, 26, 27-pentol 26-sulfate). This indicated that carp toxin is a nephro- and hepato- toxin, which could be the responsible toxin for carp bile poisoning in humans.

## 1. Introduction

Several species of fish are endowed with toxins, such as the pufferfish of the family Tetraodontidae, which possesses a potent neurotoxin, tetrodotoxin (TTX); thus, posing a serious food-hygienic threat from the standpoint of food safety. In food poisoning cases due to ingestion of aquatic organisms, the majority of causative foods are seawater fish. Among them, the most frequently reported intoxications are ciguatera and scombroid poisonings. Several of the toxins, are collectively designated as “marine biotoxins”, that have been isolated and characterized, but others remain to be studied. Although there are very few data concerning food poisonings due to ingestion of freshwater fish, two types of specific poisonings by ingestion of cyprinid fish, as the causative food, have been reported until now.

First specific poisoning, is the one that we tentatively call “Southeast Asia Type”, which is due to the swallowing of raw gallbladders of carp, *Cyprinus carpio*, and other cyprinid fish, such as grass carp, *Ctenopharyngodon idellus*, which is used as a folk remedy with a traditional belief for ameliorating rheumatism, arthritis, hypotensive, enhancement of visual acuity, cough and shortness of breath, detoxification, and for maintaining body equilibrium. A syndrome of acute renal failure and hepatitis, resulting from the ingestion of raw gallbladders of cyprinid fish, has been reported previously among people living in the rural areas of Taiwan and China, including Hong Kong. Their symptoms included characteristics such as acute renal failure, acute liver dysfunction, paralysis, convulsion, vomiting, and abdominal pain*.* [1,2,3]. Five cases of poisonings were reported with severe acute renal failure due to ingestion of raw grass carp bile, which would have been fatal if treatment had not been applied. In Japan, poisoning incidents due to ingestion of raw or dried carp *C. carpio* gallbladder have occurred sporadically, with patients showing essentially the same characteristic symptoms as described above [4,5,6,7,8,9,10,11]. The same poisonings, along with similar symptoms, were reported in Korea and the United States [12,13]. All patients denied any previous renal or hepatic diseases before swallowing the gallbladders.

The second specific poisoning is the one that we tentatively call “Kyushu Type (Japan)”, which is due to ingestion of “arai” which is sliced flesh of carp, washed with cold water and “koikoku”, which is “miso” soup of carp. Regarding the causative agent of this food poisoning, Takeda *et al*. isolated a toxin from the leftovers in a poisoning incident [14]. The toxin was fat-soluble, and highly toxic in dogs, but much less toxic in mice. This poisoning, involved 125 persons, and was accompanied with nervous function disorder, such as may be seen with TTX or paralytic shellfish poison (PSP), and which occurred only in Kyushu District, Japan, from May 1976, to October 1978, symptoms of which were slightly similar to those induced by raw carp gallbladder [14,15]. As this type of food poisoning has not occurred since then, the causative agents remain unidentified.

On the other hand, as to the causative agent of poisonings due to the swallowing of raw gallbladders of carp, the nature of the toxicity and physiological actions of ichthyogallotoxin has only been studied extensively in grass carp bile. Yip *et al*. [16] isolated a crude toxin, which was water-soluble and thermostable, from the gallbladder of grass carp, and autopsied mice that were orally administered with its bile extract, and found that the small intestine appeared red at the duodenal portion. The first signs observed in these mice were their refusal to eat and drink. Twitching of involuntary muscles and convulsions were observed before death. In addition, Chen *et al*. [17] noted that the glomerular filtration rate decreased significantly in the rats 24 h after ingestion of 0.3 mL of grass carp bile, suggesting there may be a direct influence of grass carp bile on kidney function. The bile extract caused an immediate fall in arterial blood pressure and cardiac output, and the bile salts caused a potent hemolysis *in vitro* [18]. Although toxicity has been observed after ingestion of gallbladders from different species of cyprinid fish, the similar clinical effects are in agreement with a common toxin. Though the causative substance for the poisoning due to the ingestion of carp gallbladders has been unidentified for a long time, confirmation of the structure of the sulfate ester of 5α-cyprinol [19,20] common bile alcohol with five hydroxy groups isolated from the bile of *C. carpio*, by ^1^H- and ^13^C-NMR, as well as mass spectrometry (MS), has been reported [21,22,23].

In the present paper, we intend to introduce and summarize our experimental data regarding the poisoning due to ingestion of gallbladders of carp, including isolation and basic properties of the toxin from the bile of carp, *C. carpio*, which is probably responsible for the food poisoning. In addition, we attempted to review the current information regarding the toxin and poisoning. One of the purposes of this paper is to acquaint medical doctors with food poisonings due to ingestion of cyprinid fish.

### 1.1. Case Report of the Carp Gallbladder Poisoning [24]

According to the advice of an alternative medicine practitioner, a 67 year old woman consumed the grass carp gallbladders stewed with honey as part of a meal to improve general malaise, *etc*. She developed nausea and epigastric pain two hours after ingestion and arrived at an emergency department eight hours after ingestion. Her liver tests indicated severe parenchymal injury. Her alanine aminotransferase (ALT) (formerly glutamic pyruvic transaminase (GPT)) level was 5227 IU/L (reference 1 to 21 IU/L) at 8 h, and peaked at 7340 IU/L thirteen hours after ingestion. Despite adequate hydration, she developed oliguria (2 mL/h) on day three. The diagnosis was carp gallbladder poisoning. Hemodialysis was performed for persistent fluid retention. On day 26, her renal function showed continued improvement. She was stable and followed up with renal function monitoring.

### 1.2. Case Report of the Food Poisoning due to Ingestion of Carp Dishes [15]

A 55 year old man consumed “arai” which is a sliced flesh of carp washed with cold water, and “koikoku”, which is “miso” soup of carp. He suffered from anesthesia in the mouth and speaking inarticulately three hours after ingestion. After vigorous vomitting, he developed disturbance of consciousness and unconsciousness and arrived at an emergency department. These symptoms were consistent with nervous system disorder, such as may be caused by TTX or PSP poisoning. Body temperature, blood pressure, pulse, respiration, and results of laboratory tests were normal.

## 2. Isolation of Toxic Component from Carp Bile

### 2.1. Purification of Toxic Component

**Figure 1:**
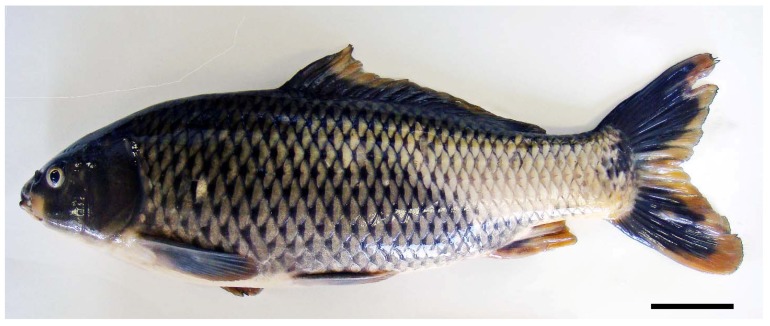
Common (Asian) carp *Cyprinus carpio*. Scale bar: 5 cm.

Gallbladders were dissected from viscera in live specimens of cultured carp *C. carpio* (Figure 1). The bile (approximately 300 mL) was collected from the gallbladders and stored at −20 °C until use. The toxin was purified and isolated from the bile according to the methods described further.

The bile was extracted with two volumes of 80% methanol under reflux for 20 min. The extract was centrifuged at 3000 rpm for 10 min. The supernatant was evaporated under reduced pressure to remove methanol, and the residue was washed several times with chloroform. After removal of the chloroform by evaporation under reduced pressure, the aqueous layer was ultrafiltered at 5 °C through a Diaflo YM-2 membrane (Amicon) with a molecular weight cut-off of 1000 Da. The filtrate was adjusted to pH 4.0 with 2 M HCl, and treated batchwise with Amberlite XAD-2 resin. The unbound fraction was lyophilized, dissolved in a small amount of methanol, and subjected to silica gel 60 (Merck) column chromatography using methanol as the eluant. The eluate was evaporated to dryness. The resulting residue was dissolved in a small amount of distilled water and loaded onto a Sep-Pak C18 cartridge (Waters). The cartridge was washed, in succession, with distilled water, 1% methanol, and 100% methanol. The toxin was eluted with 100% methanol. The eluate was evaporated to dryness and the residue was dissolved in a small amount of distilled water and subjected to high performance liquid chromatography (HPLC) on a YMC Pack D-ODS-5 column (φ 3.0 cm × 25 cm, YMC Co., Ltd., Tokyo, Japan). After washing the column with 20% methanol, the toxin was eluted using 80% methanol as the mobile phase. Fractions of 5 mL were collected at a flow rate of 7.5 mL/min. Eluates from the column were monitored by the mouse bioassay and/or thin-layer chromatography (TLC) after evaporation to dryness, followed by dissolution of the residue in water. Toxic fractions (No.18-22) were combined and lyophilized. Furthermore, The toxin was dissolved in distilled water and subjected to HPLC on a D-ODS-5 column under the same conditions, except that the flow rate was changed to 4 mL/min. Thus, the toxin was purified and finally lyophilized. Approximately 300 mg of the toxin, as white powder, named carp toxin, was obtained from 300 mL of carp bile. This toxin was soluble in water, with some foaming, and in methanol. When dissolved in methanol, the toxin did not exhibit any discernible absorption between 200 and 300 nm.

### 2.2. Thin-Layer Chromatography

TLC was performed on 10 cm × 20 cm silica gel TLC plates (Merck) with two solvent system, chloroform:methanol:acetic acid:water (65:24:15:9) and propionic acid:isoamylacetate:1-propanol:water (15:20:10:5). The toxin was detected by formation of a brown or blue spot by spraying 10% H_2_SO_4_ in ethanol or phosphomolybdic acid reagent [25,26], which is used for staining steroids, alkaloids, antioxidants, and terpenes, respectively, followed with heating for 5 min at 110 °C.

### 2.3. Mouse Bioassay for Lethal Potency

The ddY strain male mice (initial body weight of approximately 20 g) were used. Groups of five mice were separately housed in stainless steel cages. The animals were maintained on a standard laboratory pellet diet (oriental Yeast Co., Ltd., Tokyo, Japan) and distilled water *ad libitum* for at least one week before the experiments. Lethal potency was assayed by essentially the same method as that used for TTX or PSP [27]. In brief, a sample solution was injected *i.p.* into the ddY strain male mice, and their death or survival was observed for 24 h after injection. On the basis of the results obtained, the minimum lethal dose (MLD) of the toxin was estimated. Before death, usually on the fifth day, mice were anesthetized with diethyether. A small portion of liver was procured from each victim-animal, and then processed and stained for observation with light microscopy. In brief, the tissue was fixed with 10% neutral buffered formalin. Paraffin sections of 2 μm thickness were prepared and stained with haematoxylin and eosin. A group of control mice were euthanized and treated in the same manner.

### 2.4. Blood Urea-Nitrogen Analysis

Blood urea-nitrogen (BUN) in the plasma of mice was assayed by a kit of Urea N-Test Wako (Wako Pure Chemical Industries, Ltd., Tokyo, Japan) using the diacetylmonooxime (DAM) method [28]. Approximately 0.5 mL of the blood obtained from caudal veins of mice was maintained at 4 °C for 6 h. After centrifugation at 3000 rpm for 15 min, 0.2 mL of the supernatant plasma was transferred and used for BUN assay.

### 2.5. Instrumental Analysis

#### 2.5.1. IR Spectrometry

A portion of carp toxin was mixed with nujol and its IR absorption spectrum was measured with a JASCO model IR-G spectrometer.

#### 2.5.2. Mass Spectrometry

Approximately 50 μg of carp toxin was subjected to positive fast atom bombardment (FAB) mass spectrometry on a JEOL JMS-AX505W mass spectrometer equipped with a JMA-DA5000 data system at an accelerating voltage of 3.0 kV. Glycerol was used as the matrix.

#### 2.5.3. Nuclear Magnetic Resonance Spectrometry

Carp toxin (5 mg) or 5α-cyprinol (5α-cholestane-3α, 7α, 12α, 26, 27-pentol) [25,29,30], which is one of the common bile alcohols, a molecule with five hydroxy groups that was originally isolated from the bile of *C. carpio*, was dissolved in 0.3 mL deuterium oxide or methanol-*d_4_*, and placed in a test tube. ^1^H- and ^13^C-NMR spectrometric analyses, including correlation spectroscopy (COSY) and heteronuclear multiple bond correlation spectroscopy (HMBC), were performed using a JEOL JX-500 NMR spectrometer. An authentic specimen of 5α-cyprinol, which was kindly given by Dr. Takahiko Hoshita (Professor Emeritus, Hiroshima University School of Medicine), was used.

## 3. Structure of Carp Toxin

**Figure 2:**
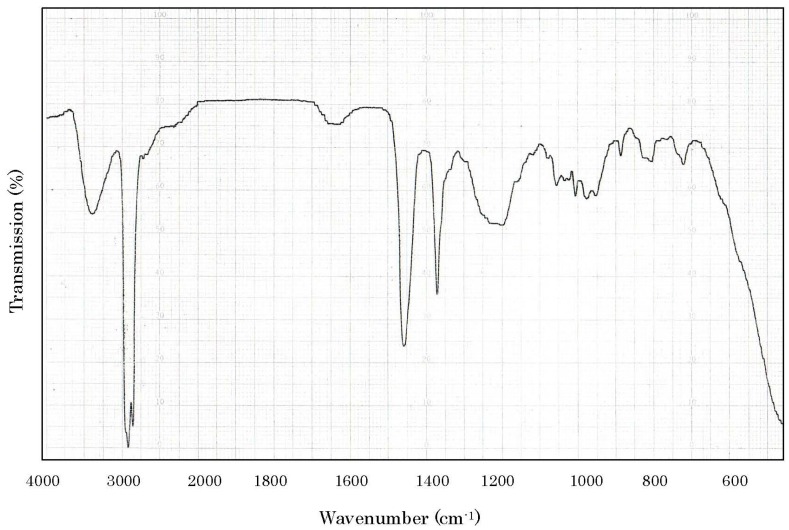
IR spectrum of the toxin isolated from gallbladders of the carp *Cyprinus carpio*. A portion of carp toxin was mixed with nujol and its IR absorption spectrum was measured with a JASCO model IR-G spectrometer.

In TLC, the toxin revealed a single spot of *Rf* 0.38 with chloroform:methanol:acetic acid:water (65:24:15:9) and that of *Rf* 0.22 with propionic acid:isoamylacetate:1-propanol:water (15:20:10:5). It formed a brown spot when detected with 10% H_2_SO_4_ in ethanol, and a blue one with phosphomolybdic acid reagent. The toxin did not exhibit any color formation with ninhydrin. IR spectrum of carp toxin is presented in Figure 2. Absorption at 3400, 1240, and 820 cm^−1^ suggested the presence of hydroxy and sulfate ester group in the molecule. The presence of sulfate was supported by the formation of a precipitate with barium chloride [20]. The IR spectral pattern was very similar to those of the compounds containing a steroid skeleton. This, along with the color development in TLC, suggested that carp toxin is a steroid-related substance possessing a sulfate ester group in the molecule.

**Figure 3:**
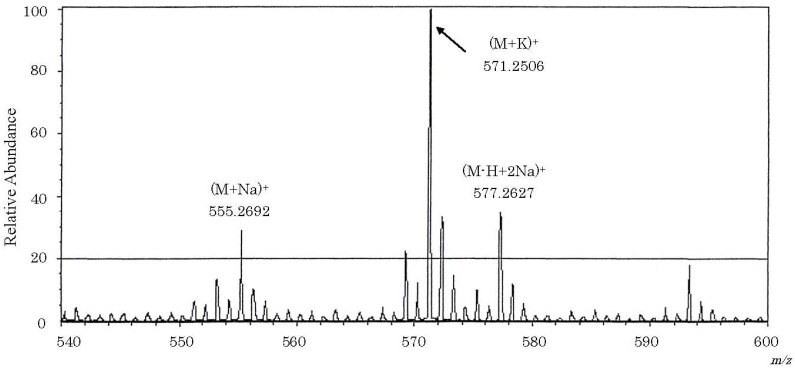
Positive FAB mass spectrum of the toxin isolated from gallbladders of the carp *Cyprinus carpio*. About 50 μg of carp toxin was subjected to fast atom bombardment (FAB) mass spectrometry on a JEOL JMS-AX505W mass spectrometer equipped with a JMA-DA5000 data system at an accelerating voltage of 3.0 kV. Glycerol was used as a matrix. Figures at several peaks denote mass numbers.

FAB mass spectrum of carp toxin is presented in Figure 3. In a scan range from *m*/*z* 540 to 600 at a high resolution (*R* = 3,000), (M + Na)^+^, and (M − H + 2Na)^+^ corresponded to values of 555.2692 and 577.2627, respectively. The fragment ion of 571.2506 was concluded to be the molecular ion of (M + K)^+^. A molecular formula of C_27_H_48_O_8_S for carp toxin was derived by this high resolution mass spectrometry.

**Figure 4:**
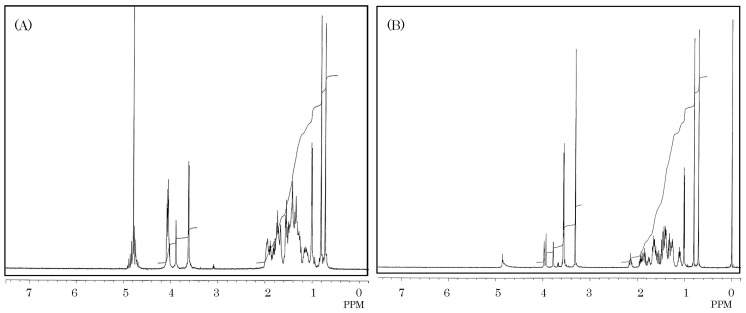
^1^H-NMR spectra of the toxin (**A**) isolated from gallbladders of the carp *Cyprinus carpio* and 5α-cyprinol (**B**). Five milligrams of carp toxin (**A**) or 5α-cyprinol (**B**) was dissolved in 0.3 mL of deuterium oxide or methanol-*d_4_*, and measured for ^1^H-NMR spectrum on a JEOL JX-500 NMR spectrometer.

^1^H-NMR spectra of carp toxin and 5α-cyprinol containing hydroxy groups at C-3, C-7, C-12, C-26, and C-27, are presented in Figure 4.

**Figure 5:**
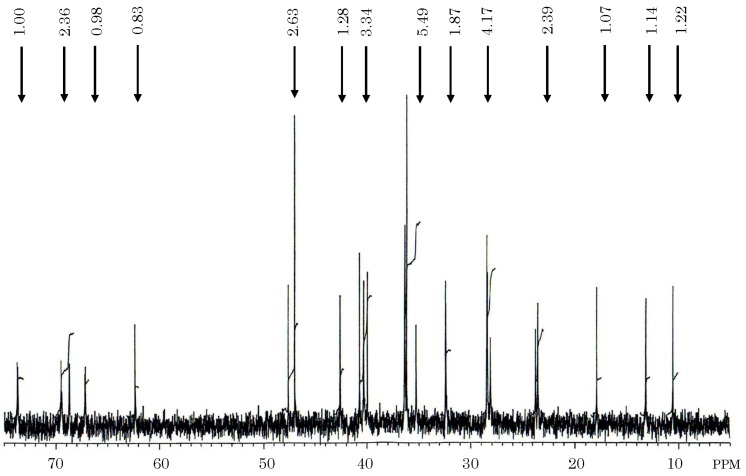
^13^C-NMR integrated spectrum of the toxin isolated from gallbladders of the carp *Cyprinus carpio*. Five milligrams of carp toxin was dissolved in 0.3 mL of deuterium oxide, and measured for ^13^C-NMR spectrum on a JEOL JX-500 NMR spectrometer. Figures at each peak denote results of the integration of the spectrum.

^1^H-NMR spectra of carp toxin was very similar to that of 5α-cyprinol. Carp toxin formed one doublet methyl (0.99 and 1.01 ppm) and two singlet methyls (0.72 and 0.80 ppm), in agreement with those of 5α-cyprinol. The total number of protons bound to oxygen-bearing carbons in carp toxin was concluded to be seven from the integrated spectrum. The seven protons in carp toxin were assigned as follows; 3.61 and 3.62 ppm (C-27:methylene), 3.88 ppm (C-3:methine), and 4.04, 4.05, 4.06, and 4.07 ppm (C-7:methine, C-12:methine, C-26;methylene). These assignments were supported by the COSY spectrum, described further. The difference in proton chemical shifts of C-26 and C-27 between both materials was explained by the presence of a sulfate ester group binding to C-26 carbon in carp toxin. The comparative carbon resonance data of carp toxin and 5α-cyprinol, measured by intensive nuclei enhancement by polarization transfer (INEPT) and distortionless enhancement by polarization transfer (DEPT) methods, elicited the presence of 10 methines, 12 methylenes, three methyls, and two quaternary carbons, providing a strong support for identity of their carbon skeletons. From the positions of chemical shifts, two methylenes (62.26 and 69.47 ppm) and three methines (67.14, 68.65 and 73.63 ppm) among them were assumed to contain oxygen-bearing carbons. These results, along with ^13^C-NMR data (Figure 5, Table 1), indicated that the carp toxin is a derivative of 5α-cyprinol, containing an additional sulfate moiety. In addition, its presence was supported by IR spectrometry of this carp toxin.

**Table 1:** ^13^C-Nuclear magnetic resonance (NMR) data of the toxin isolated from gallbladders of the carp *Cyprinus carpio* in comparison with that of 5α-cyprinol.

Carbon No.	Carp toxin	5α-cyprinol	Carbon No.	Carp toxin	5α-cyprinol
19	10.62 (q)	10.50 (q)	22	36.43 (t)	37.51 (t)
18	13.19 (q)	13.05 (q)	23	36.43 (t)	37.73 (t)
21	17.98 (q)	18.09 (q)	5	39.93 (d)	40.53 (d)
* 15	23.48 (t)	24.17 (t)	8	40.38 (d)	41.20 (d)
* 11	23.74 (t)	24.78 (t)	25	40.74 (d)	44.47 (d)
24	28.20 (t)	28.80 (t)	14	42.57 (d)	43.30 (d)
* 2	28.49 (t)	29.36 (t)	13	46.95 (s)	47.52 (s)
* 4	28.49 (t)	29.43 (t)	17	47.52 (d)	48.34 (d)
* 6	28.49 (t)	29.53 (t)	27	62.26 (t)	63.81 (t)
9	32.36 (d)	32.75 (d)	7	67.14 (d)	67.24 (d)
1	32.36 (t)	33.19 (t)	3	68.65 (d)	68.82 (d)
* 16	35.31 (t)	36.57 (t)	26	69.47 (t)	64.02 (t)
10	36.20 (s)	36.93 (s)	12	73.63 (d)	74.04 (d)
20	36.43 (d)	37.22 (d)	-	-	-

Notes: * Assignment of carbon estimated; Multiplicity was determined by INEPT and DEPT spectra.

**Figure 6:**
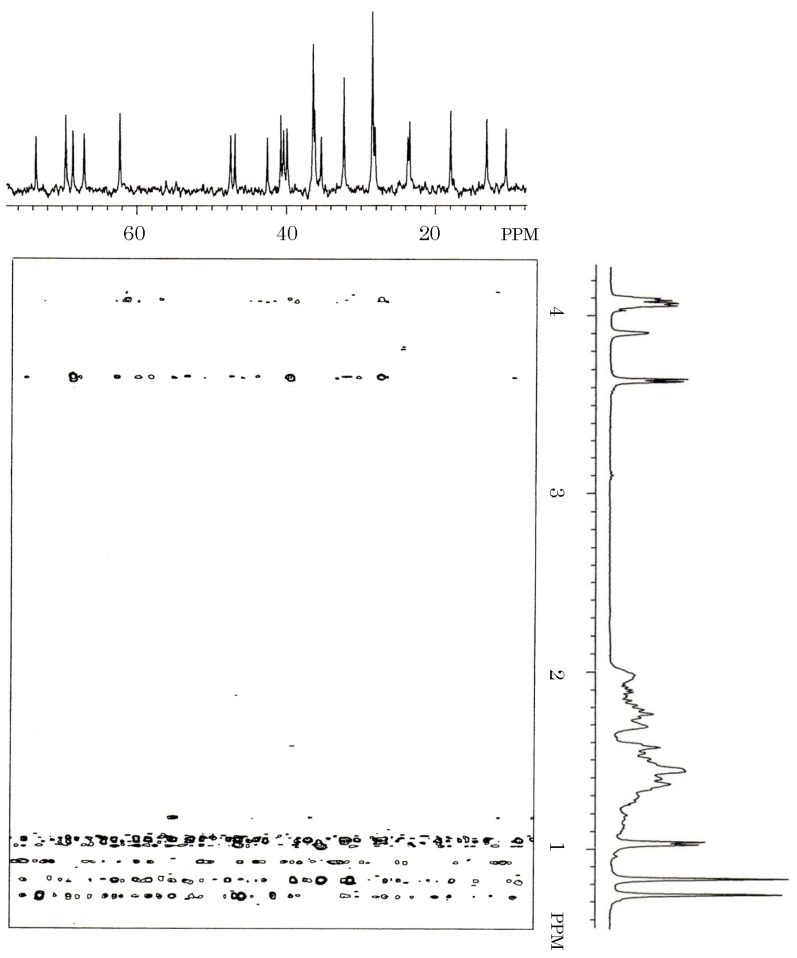
HMBC spectrum of the toxin isolated from gallbladders of the carp *Cyprinus carpio*. Five milligrams of carp toxin was dissolved in 0.3 mL of deuterium oxide, and measured for heteronuclear multiple bond correlation spectroscopy (HMBC) spectrum on a JEOL JX-500 NMR spectrometer.

HMBC spectrum of the carp toxin clearly indicated the linkage of three methylenes (28.20, 62.26, and 69.47 ppm) and one methane (40.74 ppm), and that of three methines (42.57, 47.52, and 73.63 ppm) and one quaternary carbon (46.95 ppm) (Figure 6).

**Figure 7:**
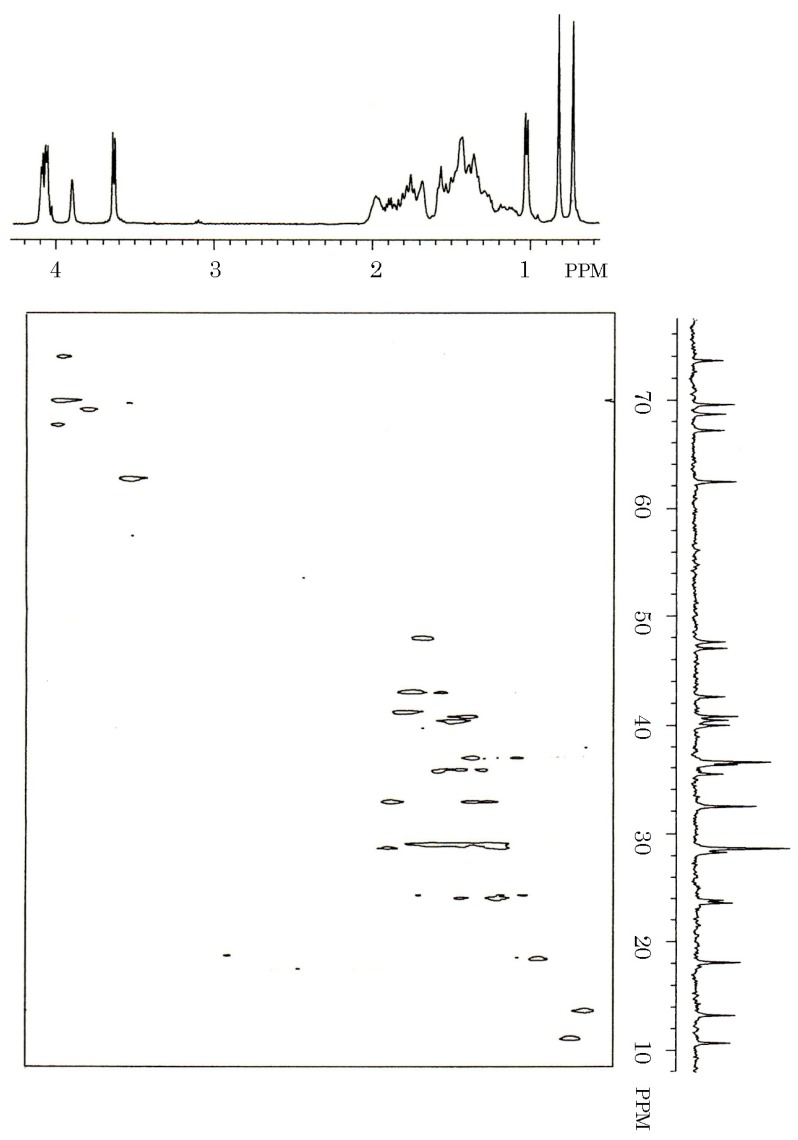
^13^C-^1^H COSY spectrum of the toxin isolated from gallbladders of the carp *Cyprinus carpio*. Five milligrams of carp toxin was dissolved in 0.3 mL of deuterium oxide, and measured for ^13^C-^1^H COSY spectrum on a JEOL JX-500 NMR spectrometer.

It indicated the linkage of two methines (32.36 and 39.93 ppm), one methylene (32.36 ppm) and one quaternary carbon (36.20 ppm), and that of two methines (36.43 and 47.52 ppm) and two methylenes (36.43 and 36.43 ppm). Along with ^13^C-^1^H COSY spectrum of carp toxin (Figure 7) and the structure of 5α-cyprinol (Figure 7), the signals at 28.20, 40.74, 62.26, and 69.47 ppm in carp toxin were assigned to C-24, C-25, C-27, and C-26, respectively.

Moreover, the linkage of C-17 (methine; 47.52 ppm), C-20 (methine; 36.43 ppm), C-21 (methyl: 17.98 ppm), C-22 (methylene; 36.43 ppm), and C-23 (methylene; 36.43 ppm) was also revealed. The linkage of carbons in carp toxin obtained by the structure analysis in the same manner provided further strong support for the similarity of its general structural with 5α-cyprinol. The chemical shifts of 21 carbons in carp toxin were assigned (Table 1). As signals at 44.47 and 64.02 ppm in 5α-cyprinol were assigned to C-25 and C-26, respectively, marked diffrences in carbon chemical shift between carp toxin and 5α-cyprinol were found for C-26 (69.47 *vs*. 64.02 ppm) and C-25 (40.74 *vs*. 44.47 ppm). The chemical shift of C-26 in carp toxin moved to downfield further than that of 5α-cyprinol, whereas chemical shift of C-25 in carp toxin moved more upfield than that of 5α-cyprinol. The 5.45 ppm downfield shift (α-effect) of C-26 in carp toxin was compatible with the suggestion that this carbon posses a sulfate ester group. In addition, the 3.73 ppm upfield shift (β-effect) of C-25 peak in carp toxin agreed with the assumed location.

**Figure 8:**
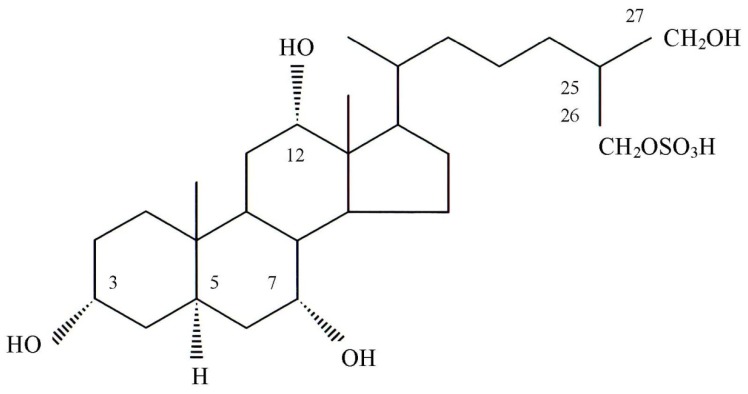
Structure of the toxin isolated from gallbladders of the carp *Cyprinus carpio*.

On the basis of these results, the structure of carp toxin was concluded to be 5α-cholestane-3α, 7α, 12α, 26, 27-pentol 26-sulfate (Figure 8). Biochemical Nomenclature of IUPAC-IUB Joint Commission was used with regard to the number of C-26 or C-27 in a cholestane skeleton [31].

## 4. Basic Properties of Carp Toxin

**Figure 9:**
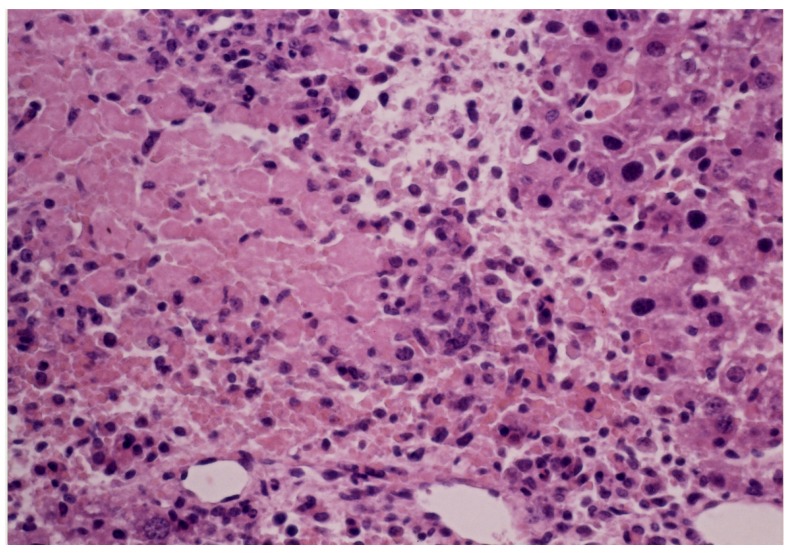
.Light microscopy (×245) of the liver from the mouse administered with the carp toxin. The tissue of liver was fixed with 10% neutral buffered formalin. Paraffin sections of 2 μm thickness were prepared and stained with haematoxylin and eosin.

The MLD of carp toxin in mice was estimated to be 2.6 mg/20 g body weight when injected *i.p.* [21]. The signs observed included paralysis convulsions of hind legs, and other neurological signs*.* In addition, the *LD_50_* values of 2.5 mg/20 g mouse was reported [32]. During intraperitoneal administration at every 24 h, with a dose of 1.0 mg, some mice died on approximately the fifth day. All of the toxin-administered mice exhibited the well-documented signs of carp poisoning, such as ataxia due to paralysis, convulsions of hind legs, and nervous disorder, before death. On the 4th day after administration of the toxin, the level of BUN increased from the initial value (16.5 ± 1.85 mg/dL) (mean ± S.D., *n* = 5) to 22.88 ± 3.18 mg/dL, indicating the dysfunction of kidney. However, mice administered with physiological saline revealed no pathological signs, and did not exhibit any signficant change in BUN level. Patients suffering due to ingestion of grass carp and/or carp gallbladders commonly developed edema and oliguria, followed by gastrointestinal symptoms, such as abdominal pain, nausea, vomiting, and watery diarrhea [2,4,5,6,7,8,9,10,11]. In most of the cases, liver dysfunction and azotemia, with a remarkable increase of BUN, were observed. Increase of BUN in the toxin-administered mice could account for acute renal failure in human patients. Liver biopsy from the mouse administered with carp toxin revealed necrosis of hepatocytes and infiltration of neutrophils (Figure 9), whereas biopsy of the control mouse liver indicated no significant changes at all. This observation image revealed findings consistent with acute toxic hepatitis. In many cases of poisonings due to ingestion of gallbladders of cyprinid fish, blood chemistry showed elevation of BUN, creatinine, ALT, and aspartate aminotransferase (AST) (formerly glutamic oxaloacetic transaminase (GOT)). Owada *et al*. [10] reported two poisoning incidents, in which patients suffered from kidney and liver dysfunctions after ingesting raw carp gallbladder. In addition, their liver biopsies revealed a mild hepatic cell cholestasis.

Similar to various esters, 5α-cyprinol sulfate is readily soluble in water and produces foam, which reminiscent of the characteristics of detergent. 5α-cyprinol is poorly soluble and did not form micelles. Considering this, Goto *et al*. [33] examined physicochemical and physiological properties of 5α-cyprinol sulfate, and reported that 5α-cyprinol sulfate is an excellent digestive detergent, surface active and formed micelles. Details of action of 5α-cyprinol sulfate on kidney and liver remains ambiguous. These signs were similar to those induced by raw carp bile in the mice. In contrast, mice administered with physiological saline revealed no pathological signs. These signs in mice resemble carp poisoning incidents, which occurred sporadically in the Kyushu district of Japan. The patients exhibited disorder of nerval functions, such as paralysis and convulsions of limbs, numbness, difficulty in speaking, risking, and walking after ingesting “arai” (sliced-washed meat) and “miso” soup of carp [14,15].

## 5. General Discussion

Our results indicated that 5α-cyprinol sulfate isolated from carp gallbladders is a nephro- and hepatotoxic substance, and could be the responsible toxin in carp bile poisoning in human. Mohri *et al*. [32] reported that essentially all of the bile toxicity can be explained by the toxicity of cyprinol sulfate, based on the *LD_50_* values. In this connection, 5α-cyprinol sulfate, which was present in gallbladder bile at high concentration (0.3 M), has been shown to be the toxic component of carp bile [34]. It was reported that the toxic effect of 5α-cyprinol sulfate on the kidney functions was more harmful than that of 5α-cyprinol [35]. The concentration of 5α-cyprinol sulfate in the carp gallbladder was reported to be 284 ± 48 mM (M ± SD, *n* = 4) [33].

Ingestion of either one, or two, or more large gallbladders, may be responsible for poisoning. Several case series, describing a cause of fish poisoning from gallbladder ingestion with resultant gastrointestinal, renal, hepatic, cadiac, and neurological toxicities, are reported. Despite the existence of a toxic substance in the gallbladders of carp and various fish species, poisoning due to the swallowing of it for the folk remedy and traditional medicine of various disorders, including arthritis and decreased visual acuity still occur in several area, particularly in rural areas of Asia. Several people are suffering from acute renal failure and hepatitis. Poisoning incidents are reported in Vietnam and India [36,37,38,39]. In Vietnam, between January 1995 and December 2000, a total of 17 patients were admitted to the hospital after consuming gallbladders from four fish species; 16 patients recovered their renal function but one patient died of fulminant hepatic failure. These poisoning incidents expands the number of intoxifications, in addition to the fresh water fish associated. The most commonly identified intoxications were the grass carp *C.* *idellus* in nine patients, and the black shark (minnow) fish *Morulius chrysophekadion* in seven patients. One patients consumed the gallbladder from the bony-lipped barb fish *Ostechilus melanopi*. Fish poison, ichthyotoxism, occurs in toxins from visceral organs (ichthyosarcotoxic), reproductive organs (ichthyotoxic), or blood (icthyohemotoxic). Causative cyprinid fish, for the poisonings due to the swallowing of gallbladders, are presented in Table 2. There are seven fishes of the order of the Cypriniformes with their bile known to be nephro- and hepatotoxic to humans. Most of the fish, such as the grass carp, silver carp, and black carp were from 0.5 to 1.2 m in length. Most of the poisoning cases developed after swallowing gallbladders from grass carp or silver carp, and their lengths were over 1 m long. The volume of bile ingested varied from 15 to 30 mL [12]. Therefore, the larger the size of the ingested Cyprinid fish is the higher is the risk of intoxication.

**Table 2:** Causative cyprinid fish of poisonings due to ingestion of gallbladder.

Common name	Scientific name	References
Carp (Common carp, Asian carp)	*Cyprinus carpio*	[4,5,6,7,8,9,10,11,12]
Grass carp	*Ctenopharyngodon idellus*	[1,2,3,11,15,16,17,23,33,34,35]
Silver carp (Silver bighead)	*Hypophthalmichthys molitrix*	[11]
Black carp (Black chinese roach)	*Mylopharyngodon piceus*	[11]
Striped bighead	*Aristichthys nobilis*	[11]
Black shark (minnow) fish	*Morulius chrysophekadion*	[33]
Bony-lipped barb fish	*Ostechilus melanopi*	[33]
Indian carp	*Labeo rohita*	[36]

In Japan, the most common causative fish species from which gallbladders are consumed is carp, family Cyprinidae [4,5,6,7,8,9,10,11]. It was proved that 5α-cyprinol sulfate is responsible for the poisoning that we tentatively called the “Southeast Asia Type”, which is due to the swallowing of raw gallbladders from carp and other cyprinid fish, such as grass carp, used as a folk remedy with a traditional belief for ameliorating rheumatism, arthritis, visual acuity, cough and shortness of breath, and for maintaining body equilibrium. In addition, it became clear that 5α-cyprinol sulfate is a nephro- and hepato-toxic substance. In the light of these toxic effects, clinicians and health centers should, not only be alert to toxic complications after ingestion of raw gallbladders of cyprinid fish, but also be aware of eating habits that may pose a risk for their patients. In this connection, in traditional Chinese medicine, the gallbladder of bears, cows, snakes, and chickens, besides those of cyprinid fish is an important folk drug. Sporadic cases of toxic hepatic and renal injury from snake gallbladder have been reported [40]. The toxic effects are dose related and there is a high mortality rate after delayed renal failure, and no direct evidence of snake bile toxin in this patient. It was reported that with regards to the effects of grass carp, snake and chicken bile on rats were less toxic than that of grass carp bile [41]. In this connection, diversity of bile salts in vertebrates, and their structural variation are discussed [42,43].

This study emphasized the importance of cyprinid fish gallbladders as an important source of ichthyosarcotoxism in the Asian population. Humans should refrain from consuming these fish indiscriminately. However, the causative agent of the poisoning, that we called it tentatively as “Kyushu Type, Japan”, which is due to ingestion of “arai” (sliced-washed meat of carp), and “miso” soup of carp remaines to be identified. Seventeen food poisonings involving 125 persons accompanied with nervous function disorder, such as TTX or PSP poisoning, occurred only in the Kyushu District of Japan from May 1976, to October 1978 [14,15]. An epidemiological survey showed that all the patients consumed raw meat and miso soup of carp. However their symptoms were similar to those induced by raw carp gallbladder, however, the causative agent of this poisoning was not viscera of carp due to gallbladders ingestion. Upon either oral or intraperitoneal administration, mice were less sensitive to the poison compared with dogs, monkeys, and cats. This purified toxic substance could be detected as a distinct blue-green spot on the plate by spraying 20% sulfuric acid, and then heating. Physical data were *m*/*z* 575 (parent mass peak) and UV λ_EtOH/max_, 220 and 282 nm. However, we extend our investigation of toxic substances related to the poisoning occurred in Kyuishu, Japan, although it is difficult to find any toxic specimens from the occurance. Search for the causative agent of this food poisoning is currently in progress by our research group. Its results will be reported in the future.
